# Distal renal tubular acidosis: a systematic approach from diagnosis to treatment

**DOI:** 10.1007/s40620-021-01032-y

**Published:** 2021-03-26

**Authors:** Sabrina Giglio, Giovanni Montini, Francesco Trepiccione, Giovanni Gambaro, Francesco Emma

**Affiliations:** 1 Medical Genetics Unit, Department of Medical Sciences and Public Health, University of Cagliari, Cagliari, Italy; 2 Fondazione IRCCS Ca’ Granda Ospedale Maggiore Policlinico, Nephrology, Dialysis and PediatricTransplant Unit, Milan, Italy; 3 Department of Clinical Sciences and Community Health, University of Milan, Milan, Italy; 4 Department of Translational Medical Sciences, University of Campania “L. Vanvitelli”, Naples, Italy; 5 Biogem Research Institute Ariano Irpino, Ariano Irpino, Italy; 6 Nephrology Department of Medicine, University of Verona, Verona, Italy; 7 Division of Nephrology, Department of Pediatric Subspecialties, Bambino Gesù Children’s Hospital, IRCCS, Rome, Italy

**Keywords:** Tubulopathy, Type 1 Distal RTA (dRTA), Rare disease, Molecular genetic test, Nephrology, Alkali treatment

## Abstract

Renal tubular acidosis (RTA) comprises a group of disorders in which excretion of hydrogen ions or reabsorption of filtered HCO_3_ is impaired, leading to chronic metabolic acidosis with normal anion gap. In the current review, the focus is placed on the most common type of RTA, Type 1 RTA or Distal RTA (dRTA), which is a rare chronic genetic disorder characterized by an inability of the distal nephron to secrete hydrogen ions in the presence of metabolic acidosis. Over the years, knowledge of the molecular mechanisms behind acid secretion has improved, thereby greatly helping the diagnosis of dRTA. The primary or inherited form of dRTA is mostly diagnosed in infancy, childhood, or young adulthood, while the acquired secondary form, as a consequence of other disorders or medications, can happen at any age, although it is more commonly seen in adults. dRTA is not as “benign” as previously assumed, and can have several, highly variable long-term consequences. The present review indeed reports and summarizes both clinical symptoms and diagnosis, long-term outcomes, genetic inheritance, epidemiology and current treatment options, with the aim of shedding more light onto this rare disorder. Being a chronic condition, dRTA also deserves attention in the transition between pediatric and adult nephrology care, and as a rare disease it has a place in the European and Italian rare nephrological diseases network.

## Introduction to dRTA physiopathology

### Renal mechanisms of acid secretion

Renal tubular acidosis (RTA) encompasses a group of disorders characterized by the inability of different segments of the renal tubule to handle bicarbonate reabsorption and/or non-volatile acid secretion thus causing impaired acid–base homeostasis. According to their pathophysiological basis, four types of RTA are typified [1].

Distal RTA (dRTA), also called type 1 RTA, is a rare genetic disorder characterized by the inability of the distal nephron to maximally increase the urinary secretion of protons (H +) in the presence of metabolic acidosis. Other forms of RTA include type 2 RTA that reflects impaired bicarbonate reabsorption in the proximal tubules and type 3 RTA that corresponds to mixed forms of type 1 and type 2 RTA. Finally, type 4 RTA is caused by aldosterone deficiency or renal tubular resistance to aldosterone. This review focuses on dRTA (type 1 RTA).

Alteration of pH homeostasis causes several cell and tissue dysfunctions. In humans, acid base homeostasis relies primarily on lungs and kidneys, which regulate volatile (PCO_2_) and non-volatile (titratable acids and NH4^+^) acid excretion, respectively. In addition, the kidneys continuously restore the systemic bicarbonate pool by reabsorbing the majority of HCO_3_^−^ filtered by glomeruli and by generating new molecules through ammoniagenesis [2].

Tubular acid secretion is achieved by several transporters in specific sections of the nephron. Sodium Hydrogen Exchanger (NHE) proteins mediate the luminal secretion of protons driven by sodium transport. This mechanism increases bicarbonate reabsorption primarily in proximal tubules and in the thick ascending limb of Henle’s loop. Secreted protons are buffered by filtered bicarbonate ions and the resulting carbonic acid (H_2_CO_3_) is dissolved in water (H_2_O) and carbon dioxide (CO_2_) by luminal carbonic anhydrases. High CO_2_-permeability of plasma membrane (favored by aquaporin-1[AQP1] along proximal tubule cells), allows CO_2_ to permeate easily into cells where it is hydrated back to H_2_CO_3_ and it dissociates into bicarbonate (HCO_3_^−^) and protons (H^+^). This process is promoted by intracellular carbonic anhydrases. Since luminal bicarbonate is the main buffer in proximal tubules and in the thick ascending limb of Henle, luminal pH does not change significantly because CO_2_ is volatile and leaves the tubular lumen.

Non-volatile acid removal occurs primarily in distal segments of the nephron by the combined action of cells lining the distal convoluted tubule, the connecting duct, and the collecting duct [3].

Type A intercalated cells (A-IC) are the main cells involved in acid secretion along the distal nephron. These cells have high plasticity [4, 5] and can convert from base- to acid-secreting phenotypes when they need to eliminate large acid loads [6]. A-IC cells are equipped with apical H^+^-ATPase (proton pump) and basolateral AE1 anion exchanger. Intracellular CO_2_ is hydrated to H_2_CO_3_ by carbonic anhydrase II. The dissociation of H_2_CO_3_ into H^+^ and HCO_3_^−^allows proton secretion through the H^+^-ATPase, which requires hydrolysis of ATP. For each proton excreted, one HCO_3_^−^ is reabsorbed into the bloodstream through the basolateral AE1, in exchange with chloride. Any alterations in the cooperation between AE1 and proton pumps impair the function of A-IC cells, leading to dRTA [7].

In addition to IC, apical H^+^-ATPases have been identified in distal convoluted tubular cells in several species [8]; however, it is unclear if they contribute to systemic or to local acid–base homeostasis. To illustrate the complexity of these processes, an additional mechanism causing type 4 RTA has recently been described in a mouse model of Gordon syndrome (pseudohypoaldosteronism type II or PHA2 [OMIM: 145260]). In this model, metabolic acidosis results from the increase in luminal bicarbonate secretion through pendrin, secondary to an increased number of B-type IC [9]. Whether other forms of dRTA may be associated to similar mechanisms is unknown. Active secretion through H^+^-ATPases can generate proton gradients across cell membranes up to 1 unit of pH during systemic acidosis. However, to maximize acid secretion, it is fundamental for urine to reach the distal nephron with strong acid buffer capacity, to quench secreted protons, allowing further secretion of free H^+^ [10]. Titratable acidity (TA) (mainly phosphate, creatinine, sulphate and to a lesser extent, urate and citrate) represents the main buffer at the level of cortical collecting ducts, while ammonium (NH_3_/NH_4_^+^) is the primary buffer at the medullary level [11]. During maximal acidification, buffers are predominantly protonated (TA^+^ and NH_4_^+^), increasing the Net Acid Excretion (NAE = TA + NH_4_^+^-HCO_3_^−^) in urine. Reduced urinary buffer capacity explains a large proportion of the impaired urinary acid secretion that is observed in chronic kidney failure.

### Clinical physiology of renal acid secretion and diagnosis of dRTA

The anion gap (AG) ([Na^+^  + K^+^−Cl^−^−HCO3^−^]; normal values: 16 ± 4 mEq/L) should be calculated in the presence of metabolic acidosis in order to gain insights on its cause, treatment and prognosis. The anion gap represents an indirect estimation of unmeasured anions, mostly proteins under physiological condition. However, it can reveal the corresponding anion of an extra-acid source:

- High AG acidosis reflects a net acid gain in the blood; the released proton is buffered by bicarbonates and the corresponding base contributes to blood electroneutrality, without any increase in chloride concentration.
- Normal AG acidosis reflects consumption/loss of bicarbonates, paralleled by an increase in serum chloride to maintain electroneutrality.

High AG acidosis is present in several conditions, including ketoacidosis, starvation, lactic acidosis, organic aciduria, uremia, and in the presence of acidic toxins. This is often associated to more severe prognosis.

Bicarbonate loss/consumption in normal AG acidosis may be secondary to gastrointestinal losses, drugs, such as acetazolamide or topiramate, or to altered renal acid base homeostasis (renal tubular acidosis).

**Fig. 1:**
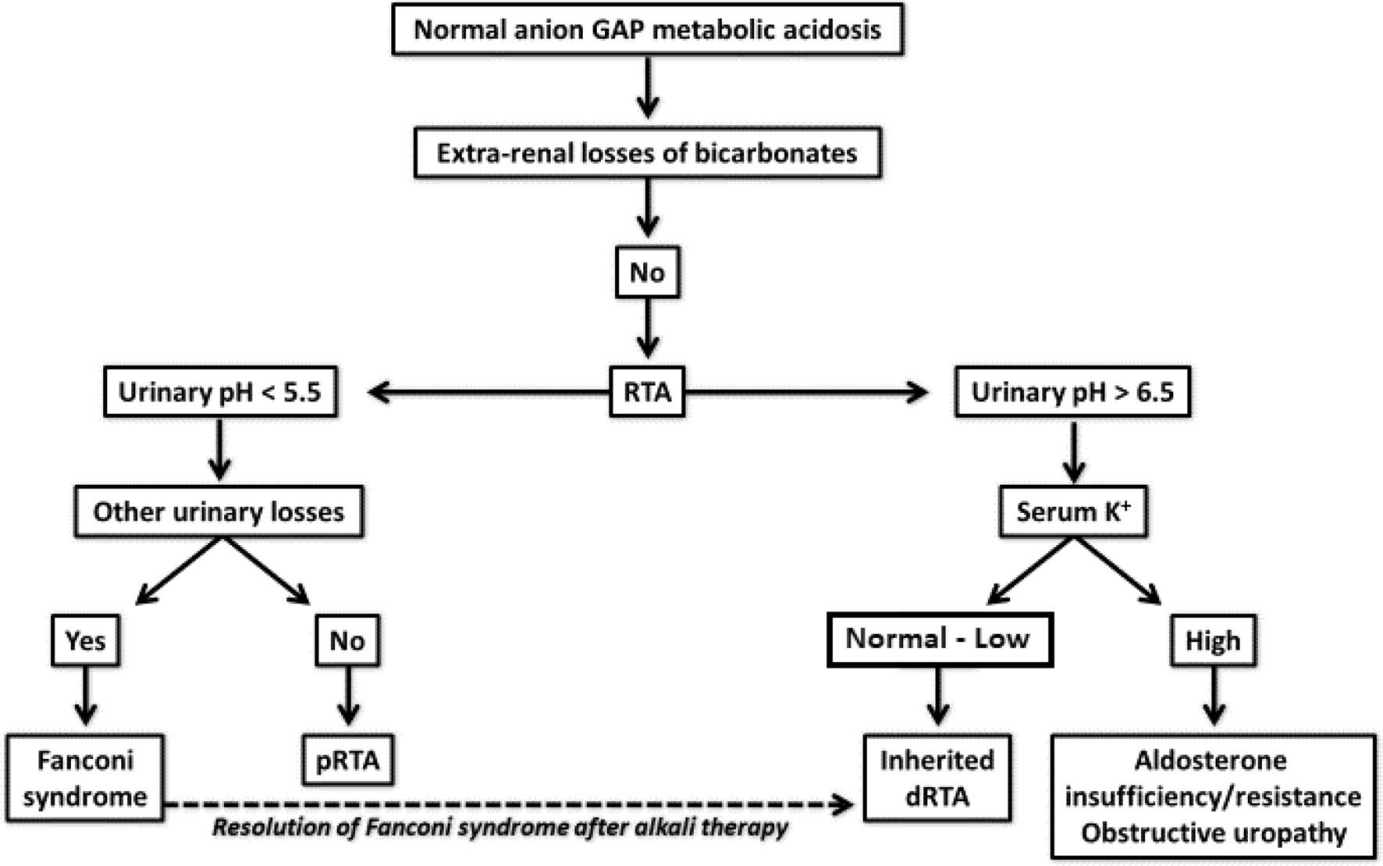
Simplified approach to diagnose patients with acidosis

Renal tubular acidosis should be suspected in all subjects with normal AG acidosis, without evidence of extra-renal bicarbonate losses (Fig. 1).

In the clinical setting, several tests have been proposed over the years to evaluate the renal acid secreting capacity, in order to differentiate different types of RTA. These tests have provided key information that have improved our knowledge of renal physiology.

The normal kidney response to systemic acidosis is to increase acid secretion and consequently to lower urine pH. In patients with normal GFR and in the absence of urinary tract infection, acidemia with an inappropriately high urinary pH (pH > 6) is suggestive of a primary defect in renal distal acid secretion.

Urinary AG (uAG) (urinary [Na^+^  + K^+^−Cl^−^] is an indirect estimation of urinary NH_4_^+^, which is difficult to measure in the clinical setting. A positive uAG indicates failure of the kidney to produce urinary NH_4_^+^ supporting the diagnosis of dRTA. Conversely, negative uAG suggests high urinary NH_4_^+^secretion, and extra-renal bicarbonate losses. Of note, uAG is not a reliable measurement in infants and neonates and does not perform well when large amounts of anions other than chloride (e.g. phosphates or ketones) are present in the urine.

Before genetic tests were introduced into clinical practice, some tests such as sodium bicarbonate load, ammonium chloride load, or the furosemide/fludrocortisone test were used to diagnose dRTA.

Occasionally, the bicarbonate loading test is still performed. This test is based on the assumption that under normal conditions, the residual buffer capacity of the HCO_3_^−^/pCO_2_couple is very limited in the distal nephron; thus, increasing HCO_3_^−^delivery to distal segments makes bicarbonate the preferred buffer for secreted protons, which increases urinary pCO_2_[12]. Measuring urinary pCO_2_is often the major technical limitation of this test [13].

Currently, the most used test is the furosemide/fludrocortisone test [14], which is particularly useful in diagnosing mild forms of dRTA. Conversely, the classic ammonium chloride acidification test is rarely performed nowadays because it is poorly tolerated and may cause severe acidosis [15]. In adults, these tests are still useful for the diagnosis of the secondary form of dRTA.

Molecular genetic tests using next generation sequencing (NGS) techniques have now replaced most of these approaches when a genetic form of RTA is suspected. These tests are cheap and fast and allow for the differential diagnosis between different genetic kidney conditions that cause systemic acidosis.

## dRTA genetics and epidemiology

dRTA is an inherited disease caused by pathogenic variants in genes involved in acid–base homeostasis in the kidney. It can be transmitted as an autosomal recessive (AR) or autosomal dominant (AD) trait. Analysis of the genetic defects causing inherited forms is fundamental to define functional consequences and genotype–phenotype correlations, and to support the exact diagnosis.

**Fig. 2:**
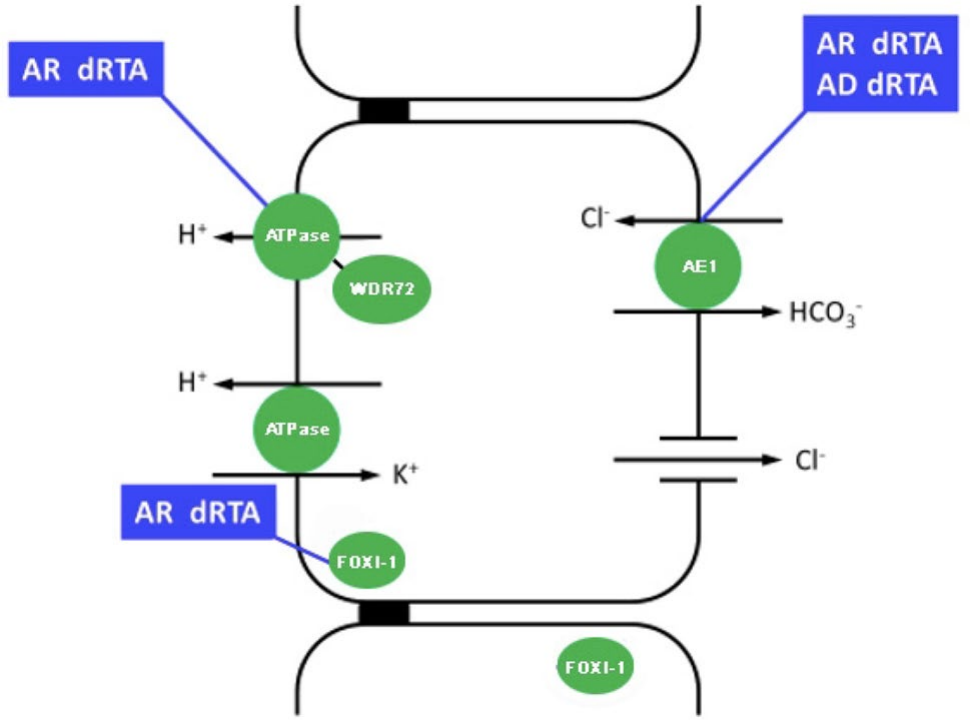
Inherited forms of type 1 RTA

This diagnosis of dRTA is established in a subject presenting biallelic causative variants in the *ATP6V0A4*, *ATP6V1B1*, *FOXI1* and *WDR72* genes, and heterozygous or (in some cases) biallelic pathogenic variants in the *SLC4A1* gene (Fig. 2).

AD dRTA is then caused by variants in the *SLC4A1* gene, encoding the basolateral Cl^−^/HCO3^−^ exchanger (AE1), necessary for HCO_3_^−^ reabsorption. The clinical manifestations of the AD form generally occur in adolescence or adulthood [16].

There are two types of AE1 protein: the shorter isoform expressed on the basolateral membrane of A-ICs that provides the main exit route for HCO_3_^−^ from cells, and the longer isoform that is expressed in red blood cells, where it is bound to other proteins of the erythrocyte cytoskeleton. In red blood cells, the AE1 protein interacts with glycophorin A, which helps ensure correct AE1 trafficking to its correct location. Therefore, pathogenic variants in the *SLC4A1* gene may cause dRTA and/or hemolytic anemia with red cell morphology anomalies.

Recessive inheritance has also been described. Recessive variants are associated with dRTA and spherocytosis or, more frequently, with spherocytosis without renal involvement, according to the domain of the protein that is mutated. dRTA and hemolytic anemia have been described mainly in Southeast Asia and have also been reported in families in the Middle East and India. Clinical symptoms usually develop in infancy or childhood [17]. This may include failure-to-thrive, polyuria, polydipsia, emesis, constipation, diarrhea, decreased appetite, and episodes of dehydration [18, 19].

The clinical and biochemical phenotype of patients with heterozygous variants in the *SLC4A1* gene is usually milder compared with that of patients with variants in other causative genes [20].

AR dRTA is usually caused by mutations in the *ATP6V0A4* or *ATP6V1B1* genes, encoding respectively for the A4 and B1 subunits of vacuolar H^+^ATPase (V-ATPase) pump expressed in A-ICs that is required for H^+^ secretion and urine acidification [18, 19, 21]. The V‐ATPase is expressed in the acid secretory A‐IC of the cortical and medullary collecting duct in the kidney, and in the epithelial cells of the endolymphatic sac of the cochlea [22].

The clinical diagnosis of dRTA associated to *ATP6V1B1* or *ATP6V0A4* genes impairment is suspected by laboratory tests showing hyperchloremic metabolic acidosis with positive urinary anion gap, and the inability of the kidney to maximally acidify the urine (urinary pH ≥ 6) (Fig. 1).

Variants in the *ATP6V1B1* or *ATP6V0A4* genes diminish the V‐ATPase proton‐secreting function and, since these subunits are also expressed in the inner ear, they can be associated with sensorineural hearing loss (SNHL)[18, 19, 21, 23].

Pathogenic variants are detected more frequently in the *ATP6V0A4* gene than in the *ATP6V1B1* gene*,* and these may include small intragenic deletions/insertions as well as missense, nonsense, or splice site variants. Furthermore, classic dRTA phenotype can also be caused by compound heterozygosity of single nucleotide variants (SNVs) with whole gene deletion/intragenic deletion in the other allele. Therefore, genomic rearrangements need to be excluded, especially in cases where only one heterozygous variant is identified and the clinical picture is strongly indicative of dRTA.

There are no mutational hot spots in these three genes. However, recurrent variants have been identified in Italian patients. In particular, the amino acid residue Arg589 is frequently mutated in the *SLC4A1* gene, p.Tyr396Thrfs*12 and p.Glu713Serfs*50 are the most frequent variants in the *ATP6V0A4* gene and p.Ile386Hisfs*56 and p.Leu81Pro are the recurrent variants observed in the *ATP6V1B1* gene. The p.Leu81Pro has been repeatedly identified in the Albanian and Apulian population, suggesting a “founder effect”.

Precise epidemiological data of the disease are still lacking. We estimated that in the Italian population, the prevalence of dRTA associated with the two recessive genes is approximately 1:600,000, allowing us to infer a carrier frequency of approximately 1/15,000, based on the Hardy–Weinberg law.

Enerbäck et al. described three patients with homozygous missense variants in the *FOXI1* gene in two unrelated consanguineous families. Patients showed early-onset SNHL and dRTA. The authors demonstrated that the mutations reduced the DNA binding affinity of FOXI1, which is critical for normal inner ear function and acid–base regulation in the kidney [24].

This gene was previously reported to be associated with deafness and an enlarged vestibular aqueduct [25]. *FOXI1* belongs to the forkhead transcription factor family, characterized by a distinct forkhead domain. It plays an important role in the development of the cochlea and vestibule and in the development of the endolymphatic system in the inner ear. It is also necessary for the expression of SLC4A1/AE1 and *ATP6V1B1* and for the differentiation of IC in the epithelium of distal renal tubules [26]. Therefore, early SNHL is highly suggestive of dRTA forms caused by pathogenic variants in the *ATP6V1B1*, *ATP6V0A4* or *FOXI1* genes*.*

In addition, whole exome sequencing (WES) of a family with dRTA has revealed compound heterozygous pathogenic variants in the tryptophan-aspartate repeat domain 72 (*WDR72*) gene in three affected siblings that also had dental abnormalities [27].*WDR72* mutations may therefore underlie dRTA cases with dental abnormalities, namely amelogenesis imperfecta, that were reported without molecular diagnosis [28].

WDR7, a human paralog of WDR72, regulates Ca^2+^-dependent exocytosis of neurotransmitters in synapses. It also interacts with the human V1 domain of the B subunit of the H^+^ATPase and co-localizes with V-ATPases in A-IC. WDR7 stimulates V-ATPase activity and mediates intracellular vesicle acidification. WDR72 may have similar functions of vesicular trafficking in IC [29].

Very recently, Hildebrandt’s group performed WES in a cohort of 17 families having 19 affected individuals with pediatric dRTA, and detected potential disease-causing mutations in three genes: *ATP6V1C2*, which encodes another kidney-specific subunit of the V-type proton ATPase (1 family), *WDR72* (2 families), and *SLC4A2* (1 family), a paralog of the known dRTA gene *SLC4A1*. Mutations in the *ATP6V1C2* and *SLC4A2* genes were further analyzed by functional assays that were conclusive for loss-of-function only for the *ATP6V1C2* variant. These results support *ATP6V1C2* as a novel dRTA gene and provide further evidence for the phenotypic expansion of *WDR72* variants [30].

Like *ATP6V0A4* and *ATP6V1B1*, *ATP6V1C2* encodes a subunit (subunit C) of the V-type proton ATPase. It is predominantly expressed in the kidney with high expression in renal IC. The affected patient of Egyptian origin showed hypokalemia, metabolic acidosis without nephrocalcinosis or deafness. He died of kidney failure at 9 years of age [30].

Nephrocalcinosis and SNHL were present in the subject with the homozygous variant in the *SLC4A2* gene that encodes the AE2 Cl^−^/HCO3^−^ exchanger, a paralog of the known dRTA *SLC4A1* gene. Since functional data have failed to provide confirmatory evidence, the role of this gene in dRTA remains hypothetical [30].

The diagnosis of dRTA is based on the observation of clinical signs and laboratory tests, but increasingly relies on genetic analyses to confirm pathogenic variants of in the classical dRTA genes, *SLC4A1*, *ATP6V1B1*, and *ATP6V0A4*. Genetic approaches with different NGS methodologies can be adapted to individual phenotypes. When phenotypic and laboratory results suggest “classic” dRTA, a multigene panel that comprises at least the following five genes could be the preferred modality: *ATP6V0A4*, *ATP6V1B1*, *FOXI1*, *SLC4A1* and *WDR72*. With the decline in costs, WES may 1 day replace genetic panel testing. This approach has allowed to establish *ATP6V1C2* as a novel recessive dRTA gene in humans and has confirmed the phenotypic expansion of recessive *WDR72* mutations from isolated amelogenesis imperfecta to syndromic amelogenesis imperfecta with dRTA. Thus, WES provides a powerful tool for identifying novel dRTA genes and, coupled with functional validation studies, helps elucidate pathogenic mechanisms of dRTA. Genome sequencing is also possible. Specific pipelines and software also allow the identification of genomic rearrangements. If panel genes or exome sequencing are not diagnostic, CGH-array and/or exome-array may be taken into consider to detect genomic rearrangements and/or (multi)exon deletions or duplications that cannot be detected by sequence analysis or bioinformatics analysis pipelines.

The NGS approach, moreover, enables to increase the detection rate of pathogenic dRTA variants, and to discriminate between other renal diseases whose clinical signs may overlap with dRTA. Molecular diagnosis is essential to provide adequate genetic counseling to patients and their families, to define prognosis, and to perform genotype–phenotype correlations.

## Pediatric dRTA: clinical aspects and long-term outcomes

Subjects with hereditary forms of dRTA may develop symptoms very early, even during infancy, including failure to thrive, vomiting, polydipsia, polyuria, feeding problems, and episodes of dehydration [31]. These symptoms improve with alkali therapy, which, if appropriate, allows normal growth in nearly all patients. From a biochemical standpoint, children typically present with hyperchloremic metabolic acidosis, hypokalemia and hypercalciuria.

Protons that accumulate during metabolic acidosis are buffered by the skeleton, inhibiting osteoblast and promoting osteoclast activity [32], which results in bone reabsorption [33], negative calcium balance, and hypercalciuria. Up to 20% of patients complain of bone pain and have bone fractures [31]. Rickets rarely causes bone deformities [34, 35], though skeletal X-ray may reveal rachitic changes, and bone densitometry shows decreased bone density [18]. In addition to hypercalciuria, proximal tubular citrate reabsorption is stimulated by acidosis, resulting in hypocitraturia [36]. Together, these urinary changes favor early onset nephrocalcinosis, and less frequently nephrolithiasis. Of note, nephrocalcinosis is always medullary; cortical nephrocalcinosis should always prompt considering other diagnoses, such as primary hyperoxaluria.

The clinical expression also depends on the type of variant. Patients with recessive AE1 pathogenic variants usually develop symptoms during infancy or early childhood. Serum potassium is low or at the lower limit of the normal range, and calciuria is usually high-normal or increased. Medullary nephrocalcinosis is present in the vast majority of patients and bone changes are frequently observed. Patients rarely present with hemolytic anemia. Patients with AD AE1 pathogenic variants usually become symptomatic later in childhood and some are not diagnosed until adulthood. Symptoms are mild; hypokalemia is frequent, while medullary nephrocalcinosis is observed in only 50% of cases and is usually moderate. These forms are thought to be secondary to a dominant negative mechanism.

Conversely, variants of the V1B1 or V0A4 subunits of the vacuolar ATPase are nearly always associated with severe phenotypes and early onset of symptoms [19]. Growth retardation develops during infancy and nearly all patients have hypokalemia, nephrocalcinosis and hypercalciuria. *ATP6V1B1* pathogenic variants are associated with the early onset of severe SNHL, whereas those in the *ATP6V0A4* gene have a more variable hearing phenotype, ranging from early to late onset SNHL [21, 37, 38].The association of dRTA with early onset SNHL is therefore not an absolute indication of mutations in the *ATP6V1B* gene. The precocity and severity of SNHL is associated with an enlarged vestibular aqueduct [37], which can also cause vertigo in some patients [39]. SNHL may require hearing aids or cochlear implants. Since it may worsen progressively during childhood it requires periodic monitoring [21]. dRTA, early-onset SNHL and nephrocalcinosis are also associated with homozygous *FOXI1* missense pathogenic variants (see above)[24].

Approximately half of all children with dRTA have some features of proximal tubular dysfunction at diagnosis, including low-molecular weight proteinuria, aminoaciduria, or phosphaturia. These regress after alkali therapy and are thought to be secondary to systemic acidosis. Of note, glycosuria is usually absent [18].

Moderate impairment of glomerular function can be present even during childhood [31]. A recent cohort study including more than 340 patients has shown that the majority of adult subjects have chronic kidney disease (CKD), and among them, approximately one quarter have CKD stage III to IV (see below).

## Complete and incomplete dRTA in adult patients

In adults, overt forms of inherited dRTA are rare and are mostly observed in pediatric patients transitioning to adulthood. In this population, dRTA is usually secondary to acquired disorders of tubular acidification or medications (see Table 1). Incomplete dRTA (idRTA) can be diagnosed during the work up of renal stone disease, nephrocalcinosis or hypocitraturia.

**Table 1:** Causes of secondary or acquired forms of dRTA

Sjogren syndrome (SS) and systemic lupus erythematosus (SLE)
Kidney transplant
Medullary sponge kidney (MSK)
Chronic obstructive uropathy
Drugs (amphotericin B, foscarnet, lithium)
Cirrhosis
Sickle cell anemia

Among the acquired forms, dRTA associated to Sjogren syndrome (SS) is the most common. During SS, the occurrence of hypokalemic metabolic acidosis, variably associated to renal stone disease, is suggestive of dRTA. dRTA can be associated with other signs of renal involvement, in particular interstitial nephritis.

From the clinical standpoint, CKD is often observed in adults with dRTA. The most recent and largest studies on dRTA have shown that CKD, particularly the more advanced stages, rarely occurs before adolescence, probably because functioning nephrons may develop compensatory hyperfiltration during childhood [19, 40].These data however, indicate that dRTA should not be viewed as a benign disease and patients should be informed of the possibility of developing CKD. dRTA should always be considered in the diagnostic work-up of adults with unexplained CKD stage 3 and 4, who typically present with metabolic acidosis. Of note, several patients included in these observational retrospective studies may not have received optimum treatment since early childhood.

Peculiar to adult patients is the diagnosis of an incomplete form of dRTA. The term “incomplete dRTA” was introduced by Wrong and Davies [15] discussing patients who did not maximally lower urine pH after an ammonium chloride test, but had no overt metabolic acidosis.

It is still uncertain whether idRTA is a distinct clinical entity or part of the dRTA spectrum. Conversion from the incomplete form to complete dRTA has been reported in few cases, suggesting the existence of a continuum [41].

Mutations in the same genes known to cause dRTA in children have also been described in adult-onset idRTA. Heterozygous truncating mutations, as well as subunit polymorphisms of *ATP6V1B1*, have been described in idRTA stone formers who had normal acidemia and urinary acidification defect [42, 43]. Imai et al. also described pathogenic variants in the*ATP6V0A4* gene in one adult patient with a late-onset form of idRTA [44]. Furthermore, we have recently reported plausible pathogenic variants of the *SLC4A1* gene in two unrelated recurrent stone formers [45].

Nevertheless, the majority of idRTA cases present neither genetic nor secondary forms [45]. This likely suggests a multifactorial etiology and/or a polygenic inheritance underlying this condition. In this respect, a role could be played by subtle renal damage secondary to previous lithotripsy treatments. Since shock waves injure tubules in the treated kidney [46], it has been hypothesized that it may result in a local acidification defect, promoting calcium phosphate lithogenesis [47].Recently, by sampling urine from single Bellini ducts, the existence of focal acidification defects has been proven in a particular stone phenotype [48].

In adults, both dRTA and idRTA are associated with low Bone Mineral Density [49]. Although still debated, the common explanation for this finding is that increased protein intake or catabolic stress may cause recurrent bouts of acid load and transient episodes of metabolic acidosis that are sufficient to cause mineral calcium losses [50, 51].Treatment with potassium citrate or bicarbonate improves bone mineral turn-over and density, and decreases calciuria in idRTA, supporting this interpretation [52]. Hypercalciuria and hypocitraturia are frequent in idRTA, thus explaining the increased risk of calcium renal stones in these patients.

Approximately 12% of so-called recurrent idiopathic calcium stone formers have idRTA [53]. Urinary acidification capacity is probably not a dichotomous but a continuous trait in stone formers, thus implicitly denying the existence of idRTA. According to this hypothesis, stone formers with idRTA correspond to the lower range of the acidification capacity [54].

Regardless of whether idRTA is or is not a specific disorder, the relevant aspect of this condition is that idiopathic calcium stone formers develop an imbalance between endogenous acid load (of dietary origin) and kidney efficiency in excreting protons [54]. This leads to hypercalciuria [55] and hypocitraturia [56], and ultimately to an increased risk of stone formation [57].These patients pass both calcium phosphate (apatite) or calcium oxalate stones, but unusually not mixed stones of these two crystalline species. Although reported [58], we have never observed nephrocalcinosis in these patients. While no specific study has been performed, at odds with overt dRTA forms, the risk of CKD and ESKD is much lower in idRTA, and is actually similar to that of idiopathic calcium stone formers.

idRTA is frequently observed also in Medullary Sponge Kidney (MSK), a disease characterized by recurrent calcium nephrolithiasis, hypercalciuria and nephrocalcinosis [59, 60]. In patients with MSK, Osther et al. [61] suggested that defective urinary acidification plays an important role in the mechanism of hypercalciuria, while Higashihara et al. [52] showed that correcting acidosis with bicarbonate reduced calciuria. We have also observed that the typical biochemical phenotype of idRTA (normal serum bicarbonate, marginally low serum potassium, less acidic fasting urine pH, hypocitraturia) is very frequently present in MSK patients and that chronic treatment with potassium citrate increases citraturia, reduces calciuria and improves bone mineral density [60]. Taken together, these observations suggest that hypercalciuria in patients with idRTA and MSK could be used as a good parameter to monitor abnormal bone turnover.

An important clinical issue in idRTA is whether this condition should be specifically investigated [50] since functional tests for its diagnosis are complex and time-consuming, and treatment of renal stone formers and MSK patients with idRTA is similar to that of patients with idiopathic calcium nephrolithiasis.

Indeed, oral administration of potassium citrate is suggested for the prevention of new stones in recurrent calcium stone formers [62]. Conversely, tests to diagnose idRTA may be appropriate in patients with osteoporosis and the suspicion of other secondary forms of dRTA (see Table 1), in whom treatment with alkali would not otherwise be administered. In these cases and in the clinical constellation of patients with calcium-phosphate containing stones, normal serum bicarbonate, but marginally low serum potassium, less acidic 24-h or fasting urine, and hypocitraturia suggest a diagnosis of idRTA, prompting a confirmatory functional tests. It has also been shown that the combination of some parameters has a good diagnostic performance (i.e. citraturia < 320 mg/24 h, 24-h urine pH > 6.2 and blood bicarbonate > 21 mEq/L has an AUC of 0.774) [54]. If the results of a furosemide-fludrocortisone test (the simplest functional test for this disorder) suggest defective tubular acidification, the association with citraturia < 360 mg/24 h and fasting urine pH > 6.0 improves the diagnostic performance of the test, without requiring the much more endeavoring test with NH_4_Cl [58].

## Treatment of dRTA

The correction of biochemical abnormalities and the prevention of complications such as nephrocalcinosis and CKD, are the goals of treatment in dRTA. The only therapy currently available is based on alkali replacement, either with citrate salts or sodium/potassium bicarbonate [53].

Alkali should be promptly prescribed to prevent osteopenia and to allow normal growth in children [63]. Maintaining normal serum bicarbonate levels raises urinary citrate, lowers the frequency of nephrolithiasis, and restores normal growth in children [64]. Several studies have demonstrated the importance of early control of acidosis starting in the first months of age to ensure normal growth. Chang et al. [65] observed for example that severe growth retardation in untreated patients improves only after maintaining appropriate alkali therapy [65]. In most children, hypercalciuria resolves when acidosis is corrected. Therefore, physicians should suspect insufficient therapy or non-compliance in dRTA children with persistent hypercalciuria.

As many as 34 different alkali formulations have been identified in a study evaluating 340 patients with dRTA [40]. Among them, 25% of patients were treated with oral bicarbonate, 42% with oral citrate and 33% with both. The median prescribed dose of alkali treatment was 1.9 mEq/kg/day. Differences in the alkali formulations may be related to the clinicians’ preference, to local practices and to the availability of different formulations in different countries. Notably, in the above mentioned cohort, the choice of alkali supplementation did not impact the achievement of satisfactory metabolic control, which was reached in only half of all patients [40].

The dose of alkali is always higher in younger patients, most likely because they produce more acid with growth [16, 18, 40]. On average, patients with vacuolar H^+^-ATPase variants need higher doses of alkali compared to patients with heterozygous mutations in the *SLC4A1* gene [18].

Potassium-containing formulations should probably be preferred in case of hypokalemia. Large doses of sodium salts should be avoided because they increase urinary calcium excretion. Potassium citrate may cause gastrointestinal tract (GI) discomfort and is sometimes not well tolerated. In these patients, a combination of potassium citrate and sodium bicarbonate often helps achieve good metabolic control.

As alkali treatment is lifelong, and requires multiple doses per day, compliance is often problematic. Recently, a new formulation, termed ADV7103, was developed. It allows a reduction in dose administration frequency to twice-a-day using a prolonged release granular formulation of K citrate (1/3) and K bicarbonate (2/3) [66]. A phase 3 multicenter, open label study including 37 patients (including dRTA adults, adolescents, children and infants) found that ADV7103 was superior in controlling metabolic acidosis. The study showed that on average, serum HCO3^−^ levels improved and were less variable. Pediatric patients seemed to benefit more from ADV7103, but the study was underpowered for sub-analyses. Patients also complained less frequently of GI discomfort, they reported improved palatability, and moreover, urinary citrate excretion increased significantly, which may have a beneficial impact in preventing nephrolithiasis, and possibly CKD [66].

A significant proportion of patients with the AR forms of dRTA suffer from SNHL. Hearing loss may be progressive, requiring monitoring during childhood, and is not reversed with alkali therapy. These patients usually require hearing aids and/or cochlear implants to improve hearing.
